# Empower Veterans Program (EVP): a chronic pain management program demonstrates positive outcomes among veterans

**DOI:** 10.1186/s12913-023-09327-5

**Published:** 2023-05-03

**Authors:** Jolie N. Haun, Christopher A. Fowler, Hari H. Venkatachalam, Michael S. Saenger, Amy C. Alman, Bridget M. Smith, Tali Schneider, Rachel Benzinger, Kevin Stroupe, Dustin D. French

**Affiliations:** 1 Research and Development Service, James A. Haley Veterans’ Hospital, 8900 Grand Oak Circle, Tampa, FL 33637 USA; 2 Division of Epidemiology, Department of Internal Medicine, University of Utah, 295 Chipeta Way, Salt Lake City, UT 84132 USA; 3 Department of Psychiatry and Behavioral Neurosciences, University of South Florida, 3515 E. Fletcher Ave, Tampa, FL 33613 USA; 4 Anesthesia Service Line, Atlanta Veterans Administration Health Care System, 1670 Clairmont Rd, Decatur, GA 30033 USA; 5 Division of Internal Medicine, School of Medicine, Emory University, 201 Dowman Dr, Atlanta, GA 30322 USA; 6 Department of Veterans Affairs, Center of Innovation for Complex Chronic Healthcare, Edward Hines, Jr. VA Hospital, 5000 South 5th Ave, Hines, IL 60141 USA; 7 Center for Health Services and Outcomes Research, Feinberg School of Medicine, Northwestern University, 633 N. St. Clair St. Suite 2000, Chicago, IL 60611 USA; 8 Departments of Ophthalmology and Medical Social Sciences, Feinberg School of Medicine, Northwestern University, 645 N. Michigan Ave. Suite 440, Chicago, IL 60611 USA

**Keywords:** Chronic Pain, Pain Management, Functional Rehabilitation, Veterans, Acceptance and Commitment Therapy (ACT), Mindful Movement, Whole health

## Abstract

**Background:**

Chronic pain is a highly prevalent health condition among veterans. Traditional pharmacological interventions present unique challenges for chronic pain management including prescription opioid addiction and overdose. In alignment with the 2016 Comprehensive Addiction and Recovery Act and VA’s Stepped Care Model to meet veterans’ pain management needs, the Offices of Rural Health and Pain Management, Opioid Safety, and Prescription Drug Monitoring Program (PMOP) funded an enterprise-wide initiative to implement a Step 3 integrated tele-pain program: Empower Veterans Program (EVP). EVP provides veterans with chronic pain self-care skills using a whole health driven approach to pain management.

**Objectives:**

The Comprehensive Addiction and Recovery Act prompted the strategic approach to offer non-pharmacological options to meet veterans’ pain management needs. EVP, a 10-week interdisciplinary group medical appointment, leverages Acceptance and Commitment Therapy, Mindful Movement, and Whole Health to provide veterans with chronic pain self-care skills. This evaluation was conducted to describe participant characteristics, graduation, and satisfaction rates; and assess pre-post patient-reported outcomes (PRO) associated with EVP participation.

**Methods:**

A sample of 639 veterans enrolled in EVP between May, 2015 and December, 2017 provided data to conduct descriptive analyses to assess participant demographics, graduation, and satisfaction rates. PRO data were analyzed using a within-participants pre-post design, and linear mixed-effects models were used to examine pre-post changes in PRO.

**Results:**

Of 639 participants, 444 (69.48%) graduated EVP. Participant median program satisfaction rating was 8.41 (Interquartile Range: 8.20–9.20). Results indicate pre-post EVP improvements (Bonferroni-adjusted p < .003) in the three primary pain outcomes (intensity, interference, catastrophizing), and 12 of 17 secondary outcomes, including physical, psychological, health-related quality of life (HRQoL), acceptance, and mindfulness measures.

**Discussion:**

Data suggest that EVP has significant positive outcomes in pain, psychological, physical, HRQoL, acceptance, and mindfulness measures for veterans with chronic pain through non-pharmacological means. Future evaluations of intervention dosing effect and long-term effectiveness of the program is needed.

**Supplementary Information:**

The online version contains supplementary material available at 10.1186/s12913-023-09327-5.

## Background

Chronic pain affects an estimated 50 million American adults. [1, 2] The effects of chronic pain are far reaching, as it often persists beyond 3-to-6 months [3] from onset and encompasses physical, psychological, and social (biopsychosocial) components. [4] As such, over a third of individuals with chronic pain experience significant disability or interference with their activities of daily living and life roles. [1, 5]

Compared to the general population, veterans are disproportionately impacted by chronic pain and associated impairments, with higher prevalence of overall (29.1% v. 19.5%) and high-impact chronic pain (9.1% v. 6.4%) [1, 6]. Furthermore, combat veterans are at greater risk of experiencing chronic pain, with prevalence as high as 81.5% among Operation Enduring Freedom and Iraqi Freedom veterans. [7] The veteran chronic pain disparity is particularly problematic because mental health (e.g., depression, anxiety, sleep) and substance use disorders are common comorbidities among people with chronic pain. [8, 9] Moreover, Ilgen and colleagues observed a dose-response relationship between pain severity and completed suicide among veterans even when controlling for demographic and psychiatric characteristics. [10] Consequently, chronic pain has been designated a high priority area within the Department of Veterans Affairs (VA) system. [11, 12]

The emphasis of screening and assessing pain as the “fifth vital sign” unintentionally led to an increased reliance on prescription opioids to treat chronic pain. The increased use of prescription opioids for chronic pain management contributed to a national opioid epidemic marked by increased rates of opioid addiction, overdose, and mortality. [13–16] In response to the opioid crisis, the Comprehensive Addiction and Recovery ACT (CARA) re-oriented VA’s mission towards interdisciplinary programs from traditional healthcare models to decrease reliance on opioid prescriptions for chronic pain management. [17] Subsequent initiatives demonstrate the VA’s commitment to conducting research on the integration of non-pharmacological interventions for chronic pain management. Non-pharmacological interdisciplinary models, consisting of integrated providers from psychology, physical therapy, nursing, etc. are a whole health (WH) and wellbeing approach to chronic pain management treatment compared to usual healthcare. [18, 19]

The pathways for experiencing and treating chronic pain are complex as nociception is not necessary for pain to occur. [20, 21] Therefore, contemporary treatment models, such as Acceptance and Commitment Therapy (ACT), target chronic pain as a biopsychosocial condition instead of treating physiological pain intensity alone. [11, 22, 23] Through the ACT model an individual explores willingness to live a high quality of life, irrespective of chronic pain discomfort, through value-driven actions (e.g., returning to work, family roles). This approach focuses on improving overall functioning, indirectly reducing pain interference. [24, 25] ACT has shown promising results when other interventions focused on pain reduction achieve limited success. [25, 26]

The importance of increasing veteran access to non-pharmacological, integrated chronic pain programs is well-established. [27–29] The Empower Veterans Program (EVP) is an interdisciplinary chronic pain rehabilitation program with the ACT model as its core behavioral therapy. EVP expands beyond ACT to integrate mindful movement (MM) and WH. An EVP pilot examined 67 graduates for pre-post EVP improvements on clinical outcomes including pain intensity, catastrophizing, and health-related quality of life (HRQoL). [30] Clinical improvements were in the medium-to-large effect size range, with high satisfaction rates. [30] These findings were comparable to other large-scale non-pharmacological integrated chronic pain programs conducted in the VA system. [27, 31] Early EVP results indicate this program as a viable option to provide veterans with accessible and sustainable integrated non-pharmacological rehabilitation for chronic pain. [30, 32]

This paper follows a cohort of Veterans who completed EVP at a large VA Medical Center in the southeast who were unresponsive to and/or declining medications, procedures, and sequential monotherapies such as Cognitive-Behavioral Therapy (CBT) and Physical Therapy. In this paper we report on pre-post changes in patient-reported outcomes (PRO). Pain-related measures represent the primary outcome; physical, psychological, social, HRQoL, acceptance, and mindfulness measures represent secondary outcomes. The objective of this paper is to report on the preliminary effectiveness of EVP in this cohort and to describe the fit of EVP in the VA’s overall mission to provide innovative non-pharmacological strategies for chronic pain management and functional rehabilitation.

## Methods

### EVP intervention

EVP cohorts range from 3 to 20 veterans that meet in-person weekly, for 10 consecutive weeks. Each meeting comprises of three, one-hour group sessions incorporating ACT, WH, and MM evidence-informed therapies. A total of 30 modules are facilitated by trained professionals (ACT – Psychologists; MM – Physical Therapists; WH – Chaplains) and incorporate topic areas including values and personal choices, mindful awareness training to notice thoughts and stressful emotions, safe movements, sleep, nutrition, and practices of compassion and gratitude. During each session, facilitators utilize scripts to engage participants in experiences that reinforce the main objectives presented in each module. Veteran activities included worksheet assignments, EVP Mindful Awareness and Compassion exercises on CD, handouts, video clips, group discussions, self-observations, and mobility exercises (simplified Yoga, Tai Chi). At-home practices are recommended to apply EVP strategies to daily life. In addition to program attendance, an EVP Chaplain conducts individual weekly follow-up coaching calls using motivational interviewing informed reflection. Though the program uses a standardized approach to optimize fidelity, EVP is designed to deliver a personalized experience to help veterans to confidently live fuller lives, usually with less pain, based on their individual values. A breakdown of EVP sessions by therapy type is shown in Table 1.

**Table 1:** Sample 10-Week Curriculum for the Empowered Veterans Program

Week	EVP Core Components and Weekly Objectives		
	EVP ACT	EVP MM	EVP WH
1	• Introductions • Reviewing group guidelines • Overview of ACT	• Introductions • Pain & the Brain Part 1	• Introductions • Overview of WH
2	• Exploring personal values and life purpose	• Pain & the Brain Part 2 • Neutral Spine	• Introducing Mindful Awareness • Power of the Mind
3	• Metaphors exploring Psychological Flexibility	• Motion is Lotion Exercises (MILES) 1 • Hand motion	• Mindfulness practices • Food and Drink
4	• Noticing added suffering from current avoidance/ coping strategies	• MILES 1 & 2 • Head and eyes motion	• Mindfulness practices • Recharge/Sleep
5	• Defusion; Tricks of the mind	• MILES 1–3 • Feet and foot motion	• Observer Self practice • Choice of gratitude
6	• Cycle: Behaviors, Thoughts, and Emotions	• MILES 1–4 • Core motion • Computer workstation	• Self-Compassion practice • Choice of Kindness
7	• Committed Action	• MILES 1-5 • EVP Tai Chi (Part 1)^1^	• Self-Compassion practice • Choice of active listening in relationship building
8	• Acceptance/Willingness	• MILES 1–5 • EVP Yoga^1^	• Self-Compassion practice • Considering choice of forgiveness
9	• Maintaining progress	• MILES 1–5 • EVP Tai Chi (Part 2)^1^	• Self-Compassion practice • Finding meaning in suffering
10	• Values declaration • Graduation	• EVP Tai Chi • Graduation	• Whole Body Scan • Graduation

^1^Tai Chi and Yoga not provided by certified instructors but consistent with the MM portion of EVP’s programmatic modalities.

ACT = Acceptance and Commitment Therapy; EVP = Empower Veterans Program; MM = Mindful Movement; WH = Whole Health.

### Design and sample

**Fig. 1:**
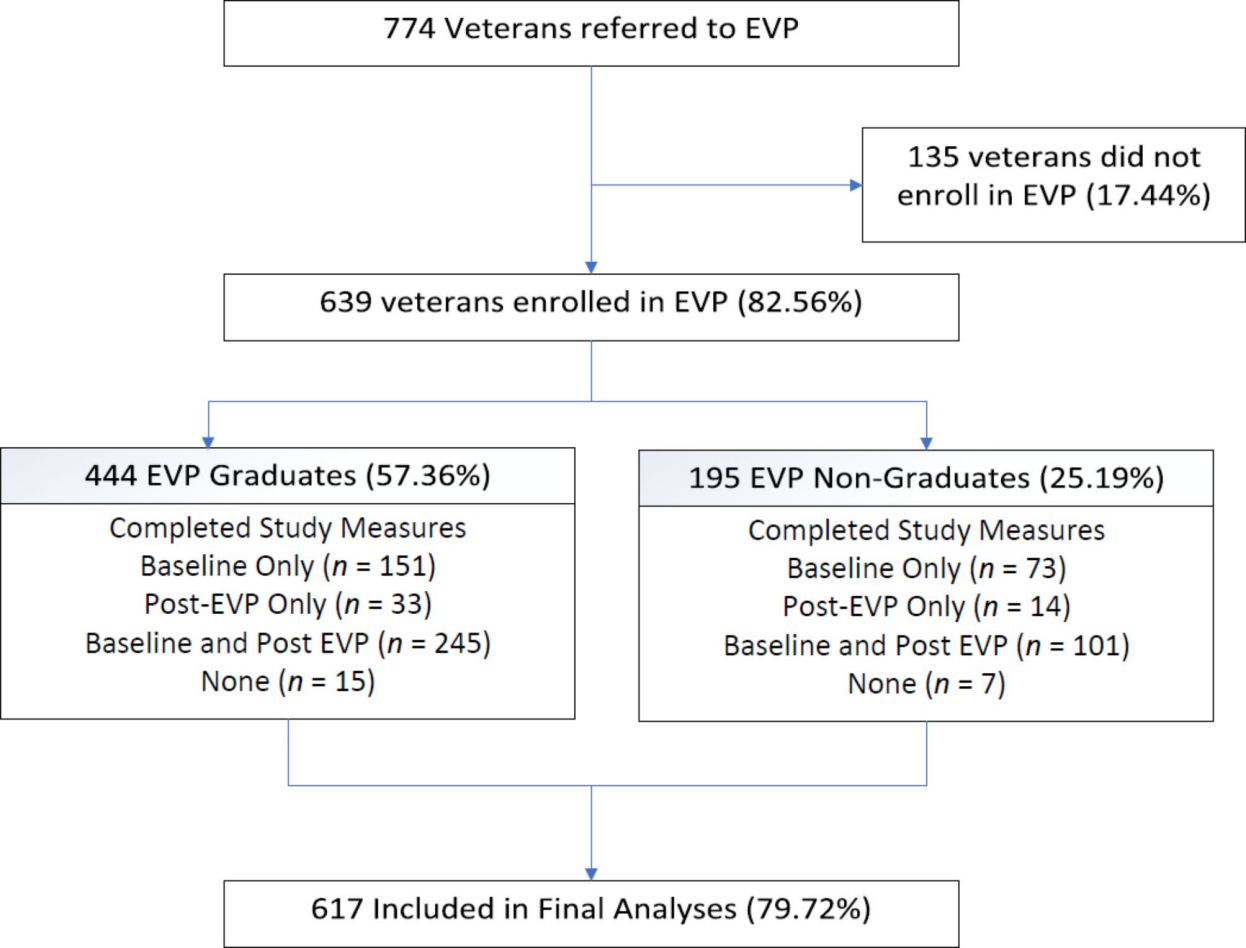
Empower Veterans Program (EVP) recruitment from 2015 to 2017

A within-participants pre-post design was used to examine PRO changes from week 1 (baseline) and the end of the program at week 10 (post-EVP). A cohort of 774 veterans with chronic pain were referred to EVP between May 2015 and December 2017. In total, 639 of these veterans (89.20%) enrolled in EVP and participated during this evaluation time period; final primary analyses included 617 veterans (79.72%) that completed PRO measures for at-least 1 time point and were included in final analyses. Figure 1 presents a flow diagram of veteran sampling.

### Data collection

Data was collected at a VA medical center in the southeastern United States. Standardized PRO measures were administered at the beginning of week 1 (baseline) and again at week 10 (post-EVP). Participant data were stored in Excel version 2201 spreadsheets on a secure drive behind the VA firewall. The data collection protocol was reviewed for ethical compliance by the Emory University Institutional Review Board and deemed non-research and was conducted as a quality management project. All veterans provided informed consent prior to their participation.

### Outcome measures

Given the preliminary quality management nature of this evaluation, exploratory measures including EVP graduation status and satisfaction rates were examined. To operationalize EVP graduation rate, we adopted the clinical requirements for the program. Specifically, participants that graduated from EVP completed ≥ 80% of sessions (≥8/10) and non-graduates completed less than ≤ 80% of EVP sessions (≤ 7/10). Primary pain-related outcomes (intensity, interference, catastrophizing), as well as secondary outcomes including physical health (functioning, fatigue), psychological health (depression, anxiety, sleep disturbance), social, HRQoL (physical, psychological, social, environmental), acceptance (activities engagement, pain willingness), and mindfulness were examined using validated PRO measures (Table 2).

**Table 2:** Patient-reported outcome measures administered to assess the Empower Veterans Program (EVP)

Scale	Construct	Description	Items	Scale
**Pain**				
Pain, Enjoyment, and General Activity (PEG) [33]	Pain Intensity	Pain intensity and interference with life enjoyment and general activity in the past week	3	0 – *no pain at all*, 10 – *worst possible pain*
PROMIS-29 – Pain Interference subscale [34]	Pain Interference	The impact of pain on daily functioning.	4	1 – *not at all*, 5 – *very much*
Pain Catastrophizing Scale (PCS) [35]	Pain Catastrophizing	Maladaptive and exaggerated beliefs “toward actual or anticipated” pain experiences” (p. 602).	13	0 – *not at all*, 4 – *all the time*
**Physical**				
PROMIS-29 – Fatigue subscale [34]	Fatigue	Physical fatigue.	4	1 – *not at all*, 5 – *very much*
PROMIS-29 – Physical Functioning subscale [34]	Physical Functioning	Perceived physical capability to engage in daily activities (e.g., self-care, endurance).	4	1 – *unable to do*, 5 – *without any difficulty*
**Psychological**				
PROMIS-29 – Anxiety subscale [34]	Anxiety	Anxiety symptom severity.	4	1 – *not at all*, 5 – *very much*
Patient Health Questionnaire-9 (PHQ-9) [36]	Depression	Depression symptom severity.	9	0 – *not at all*, 3 – *nearly every day*
PROMIS-29 – Sleep subscale [34]	Sleep Disturbance	Difficulty falling and staying asleep.	4	1 – *not at all*, 5 – *very much*
**Social**				
PROMIS-29 – Social subscale [34]	Social Health	Social roles (e.g., family, occupational) and activities (e.g., leisure)	4	1 – *always*, 5 – *never*
**HRQoL**				
World Health Organization Quality of Life Scale Brief Version (WHOQoL-BREF) [37]	Physical HRQoL	Mobility, daily activities, functional capacity, energy, pain, and sleep.	26	1–5^+^
	Psychological HRQoL	Self-image, negative thoughts, positive attitudes, self-esteem, mentality, learning ability, memory, concentration, religion, and mental status.		
	Social HRQoL	Personal relationships, social support, and sex life.		
	Environmental HRQoL	Financial resources, safety, health and social services, physical living environment, opportunities to acquire new knowledge and skills, recreation, general environment, and transportation.		
**Acceptance**				
Chronic Pain Acceptance Questionnaire (CPAQ) [38]	Activity Engagement	Engagement in life activities despite experiencing pain.	20	0 – *never true*, 4 – *always true*
	Pain Willingness^*^	Willingness to experiences pain without attempts to control it.		
**Mindfulness**				
Five-Facet Mindfulness Questionnaire (FFMQ) [39]	Acting	Acting with awareness in the moment.	39	0 – *never or rarely true*, 4 – *very often or always true*
	Describing^*^	Describing how we label experiences to ourselves and others.		
	Non-Judgement^*^	Non-Judgement despite self-criticism.		
	Non-Reactivity	Non-Reactivity of negative thoughts and emotions while accepting their presence.		
	Observation	Observation of our sensory experiences to focus our attention on meaningful stimuli.		
**Program Satisfaction**				
Pain Outcomes Questionnaire – Veterans Affairs (POQ-VA) [40]	EVP Satisfaction	Satisfaction with the EVP administered at week 10 (post-EVP).	5	0–10^+^
**Covariates**				
POQ-VA [40]	Demographics	Age	5	Continuous
		Gender		Male v. Female
		Race		White v. Non-White
		Service-Connected Disability		Yes, No
		Religion		Nominal

^*^Reverse-scored for interpretive consistency

^+^Item-level response options vary by domain

PROMIS = Patient Reported Outcome Measurement Information System

HRQoL = Health-Related Quality of Life

### Statistical analysis

#### Preliminary analyses

Means and standard deviations were used to describe continuous data. Categorical data were presented using frequencies and percentages. The median (*mdn*) and interquartile range (*IQR*) were used to describe program satisfaction which was negatively skewed. Age was mean-centered and race was dichotomized (white/non-white) for analyses. Separate logistic regression models were used to compare demographic characteristics between survey responders and non-responders at each time point (baseline, post-EVP). Survey responders were defined as responding to at-least one survey at either specified time point. Mean scale scores were calculated and used for analysis for each PRO if ≥ 70% of scale items were completed at a given time point. [41] Of note, The brief World Health Organization Quality of Life Scale (WHOQoL-BREF) was not scored if ≥ 20% of data is missing as instructed in its manual. [42] Of note, a standardized WHOQoL-BREF scoring approach was used. [42] With the exception of social HRQoL, using standardized scores in the primary analyses produced non-positive definite matrices which produce unreliable results. Mean scores were used for the remaining HRQoL scales which resulted in model convergence.

Demographic characteristics including age, gender, and race were examined as predictors of graduation rate which is consistent with disparities in intervention engagement noted in previous research. [43] No a priori hypotheses were specified for the effects of demographic characteristics on PRO measures. Analysis of participant demographics were conducted at each time point to determine which characteristics to include as covariates in the primary analyses. Pearson correlations were calculated to examine the associations between age and PRO measures. Independent samples *t*-tests and one-way analysis of variance (ANOVA) models were conducted to examine the associations between categorical demographic characteristics and PRO measures. For preliminary analyses, to determine possible covariates for the primary analyses a *p*-value of 0.05 was used to determine statistical significance.

#### Primary analyses

A logistic regression model was used to examine whether demographic characteristics predict the odds of participants graduating from EVP. The Hosmer Lemeshow test was used to assess goodness of fit for the logistic regression model. A Mann-Whitney *U*-Test was used to examine whether post-EVP satisfaction rates were significantly higher among EVP graduates versus non-graduates.

To assess pre-post EVP changes, separate linear-mixed models (LMMs) were fit for each PRO measure. The LMMs used an auto-regressive covariance matrix with maximum likelihood estimation. Mixed models can handle unbalanced data by making use of available information when outcome data is missing at a time point, thus preserving sample size. [44, 45] To establish base models to test hypotheses, random intercepts were fit to each model to account for the within-participant correlations between baseline and post-EVP measurements. All base models included time (baseline, post-EVP), graduation (graduates, non-graduates), and their interaction as fixed effects. Non-significant interactions were dropped from the model because of potential multicollinearity impacting standard errors (inflating). Independent variables were analyzed using planned contrasts. For the time variable, baseline was coded positively (+ 0.5) and post-EVP scores were coded negatively (-0.5). For interpretation of this coding scheme, a negative time coefficient indicates a decrease from baseline to post-EVP while a positive coefficient shows an increase. For graduation, EVP graduates were expected to have more positive PRO outcomes and were coded positively (+ 0.5) while non-graduates were expected to have worse PRO scores and thus, were coded negatively (-0.5). When demographic covariates were significantly associated with PRO measures (see preliminary analyses), the specific demographic variable and its interactions with time and graduation were also examined as fixed effects. In such models, non-white individuals and females were coded negatively (-0.5) consistent with previous research indicating worse pain presentations compared to white individuals and males (coded + 0.5), respectively. [43, 46] For analyses that examined race as a predictor variable, missing race cases were excluded from analyses (*n* = 6). Significant covariates were retained while non-significant covariates were dropped from respective models. Overall fit for model comparisons was examined using Akaike’s Information Criterion. To account for family-wise error rate associated with multiple comparisons, Bonferroni corrections were applied to the 20 primary and secondary outcomes. A conservative Bonferroni-adjusted *p*-value of (0.05/20 = 0.0025) was used to determine statistical significance for analyses of all primary and secondary outcomes. Analyses were conducted using SPSS version 27. Standardized mean differences (*SMD*) for correlated samples were calculated using a Microsoft Excel macro developed by Lakens. [47] The macro first calculates Cohen’s *d* and then applies a Hedges’ *g* correction to minimize positive bias for the sample estimate. [47] Effect sizes for Cohen’s *d* and Hedges *g* were adopted from a recent meta-analysis of chronic pain interventions that aim to enhance positive affect rather than reduce negative affect (e.g., ACT, mindfulness): ≤0.32 = small; 0.33-0.55 = moderate; ≥0.56 = large [48].

## Results

### Sample characteristics

EVP participants (*n* = 639) ranged from 24 to 88 years old (*m* = 56.15 ± 10.75), were primarily male (70.89%), Black or African American (74.65%), identified as Christian (76.53%), and had VA service-connected disability (81.06%). Participant demographic characteristics by graduation status or EVP program completion are presented in Table 3.

**Table 3:** Demographic information for Empower Veterans Program participants for entire sample by graduation status

Characteristics	Graduates (*n* = 444)	Non-Graduates (*n* = 195)	Total (*n* = 639)	*p*-value^a^
Age (years),				
*m* ± *sd*	57.26 ± 10.29	53.61 ± 11.37	56.15 ± 10.75	< 0.001
Gender, *n*(%)				
Female Male	120 (27.03%) 324 (72.97%)	66 (33.85%) 129 (66.15%)	186 (29.11%) 453 (70.89%)	0.081
Race, *n*(%)				
Asian/Pacific Islander Black/African American Native American/Alaskan Native White/Caucasian Biracial/Multiracial Declined to Respond Missing	3 (0.68%) 349 (78.60%) 0 (0.00%) 79 (17.79%) 1 (0.23%) 10 (2.25%) 2 (0.45%)	2 (1.03%) 128 (65.64%) 1 (0.51%) 50 (25.64%) 1 (0.51%) 9 (4.62%) 4 (2.05%)	5 (0.78%) 477 (75.65%) 1 (0.16%) 129 (20.19%) 2 (0.31%) 19 (2.97%) 6 (0.94%)	.018^b^
Service Connection, *n*(%)				
Service Connected Not Service Connected Missing	360 (81.08%) 83 (18.69%) 1 (0.23%)	83 (81.03%) 33 (16.92%) 4 (2.05%)	518 (81.06%) 116 (18.15%) 5 (0.78%)	0.906
Religion, *n*(%)				
Theist Christian Theist Non-Christian Atheist/Agnostic/Unidentified Missing	348 (78.38%) 14 (3.15%) 81 (18.24%) 1 (0.23%)	141 (72.31%) 9 (4.62%) 41 (21.03%) 4 (2.05%)	489 (76.53%) 23 (3.60%) 122 (19.09%) 5 (0.78%)	0.884

*n* = number of observations; *m* = mean; *sd* = standard deviation.

^a^ Between-group differences for Empower Veterans Program graduates by demographics characteristics.

^b^ Based on white versus non-white comparison.

### Preliminary analyses

Of the 639 participants, 570 (89.20%) provided baseline data, whereas 393 (61.50%) responded post-EVP, and 346 (54.15%) responded at both time points. Separate logistic regression models found no demographic differences between responders and non-responders at baseline (*p* = .464-.986) or at post-EVP (*p* = .169-.899).

Pearson correlations revealed no significant associations between age and any PRO outcomes (*p* = .055-0.975). Independent-samples *t*-tests revealed that baseline Patient-Reported Outcome Measurement Information System (PROMIS-29) sleep disturbance scores were 0.211 points higher among females than males (*p* = .020). No other differences were observed by gender (*p* = .079-0.961) or service-connected disability status (*p* = .078-.980). One-way ANOVA models did not reveal significant differences in PRO between religious groups (*p* = .067-0.978). Due to observed gender differences on PROMIS-29 sleep scores, gender was examined as a possible predictor of sleep disturbance.

Several potential differences were observed between white and non-white participants. On average, white participants had lower baseline scores on Chronic Pain Acceptance Questionnaire activity engagement (*p* = .013), WHOQoL-BREF psychological subscale (*p* = .040), as well as three Five-Facet Mindfulness Questionnaire (FFMQ) scales including acting with awareness (*p* = .011), describing self-experiences (*p* = .022), and non-reactivity to negative thoughts and emotions (*p* = .033). On average, white participants also had higher PROMIS-29 anxiety scores at baseline (*p* = .049) and post-EVP (*p* = .047). No other race differences were observed (*p* = .10-0.86). This pattern of results indicated that white participants may have experienced more negative outcomes than non-white participants, as such race (white v. non-white) was chosen as a possible predictor in each PRO model comparison.

### Primary analyses

#### Graduation status

In total, 444 participants (69.48%) graduated from EVP. A logistic regression model was used to examine whether graduating from EVP was associated with demographic characteristics. The Hosmer Lemeshow test indicated that this model adequately fit the data *χ*^*2*^(8) = 5.041, *p* = .753. Participant age (*p* < .001) and race (*p* = .008) were significantly associated with graduation status, but not gender (*p* = .399), religion (*p* = .676), or service-connected disability (*p* = .981). Each one-year increase in age was associated with a 3.1% increase in the odds of being an EVP graduate versus a non-graduate, *OR* = 1.031, 95% confidence interval (*CI*; 1.014, 1.049). White participants were 43.4% less likely to graduate from EVP than non-white participants, *OR* = 0.566, 95% *CI* (0.372, 0.862), but the effect size for this model was small, Cox and Snell *R*^*2*^ = 0.036.

#### EVP satisfaction

Post-EVP satisfaction rates were examined using the Pain Outcomes Questionnaire-VA 0–10 scale. EVP satisfaction rate scores had a range from 3.20 to 9.80. The median EVP score was 8.41 points with an *IQR* from 8.20 to 9.20. A Mann-Whitney *U* test found that overall, post-EVP satisfaction rates did not differ between graduates and non-graduates, *U* = 5063.50, *p* = .081.

#### Patient-reported outcomes

The PRO models primarily focused on the predictors included in the base models including fixed main effects of time, graduation, and their interaction. None of the models produced a significant time x graduation interaction effect after accounting for the Bonferroni-adjusted significance threshold. Non-significant time by graduation interaction terms were excluded from the final models because of potential multicollinearity inflating standard errors. Results from the LMMs are presented in Table 4.

**Table 4:** Fixed effects estimates for patient-reported outcome measures

Outcome	Fixed Effects	*p*-value		95%*CI*				Outcome	Fixed Effects	*p*-value	95%*CI*	
	β ± *SE*			*LB*		*UB*			β ± *SE*		*LB*	*UB*
**PAIN**								**Social**				
*Intensity*								Time	-0.234 ± 0.041	< 0.001^*^	− 0.315	-0.153
Time^a^	-0.305 ± 0.061		< 0.001^*^		-0.426		-0.184	Graduation	-0.034 ± 0.078	0.664	-0.186	0.119
Graduation^b^	0.096 ± 0.128		0.754		-0.155		0.347	Race^c^	-0.189 ± 0.089	0.034	-0.364	-0.015
*Interference*								**HRQoL**				
Time	-0.397 ± 0.044		< 0.001^*^		-0.483		-0.311	*Physical*				
Graduation	0.000 ± 0.075		0.998		-0.147		0.147	Time	1.132 ± 0.116	< 0.001^*^	0.902	1.361
*Catastrophizing*								Graduation	-0.238 ± 0.225	0.291	-0.903	0.205
Time	-0.404 ± 0.045		< 0.001^*^		-0.493		-0.316	*Psychological*				
Graduation	0.052 ± 0.092		0.570		-0.128		0.232	Time	0.887 ± 0.113	< 0.001^*^	0.663	1.110
**Physical**								Graduation	-0.054 ± 0.291	0.851	-0.626	0.516
*Fatigue*								Race	0.797 ± 0.331	0.016	0.147	1.448
Time	-0.296 ± 0.047		< 0.001^*^		− 0.388		-0.205	*Social*				
Graduation	0.021 ± 0.084		0.807		-0.145		0.186	Time	6.128 ± 0.961	< 0.001^*^	4.326	8.019
*Physical Function*								Graduation	0.907 ± 2.101	0.666	-3.221	5.034
Time	-0.149 ± 0.036		<0.001^*^		-0.219		-0.078	*Environmental*				
Graduation	0.080 ± 0.074		0.282		-0.066		0.225	Time	0.569 ± 0.114	< 0.001^*^	0.344	0.793
**Psychological**								Graduation	-0.518 ± 0.269	0.055	-1.048	0.011
*Anxiety*								**Mindfulness**				
Time	-0.250 ± 0.044		< 0.001^*^		-0.336		-0.164	*Acting*				
Graduation	-0.041 ± 0.094		0.666		-0.226		0.145	Time	0.089 ± 0.036	0.014	0.018	0.160
Race	-0.277 ± 0.108		0.011		-0.489		-0.065	Graduation	0.118 ± 0.078	0.132	-0.036	0.271
*Depression*								Race	0.234 ± 0.089	0.009^*^	0.059	0.409
Time	-0.315 ± 0.029		< 0.001^*^		-0.372		-0.257	*Describing*				
Graduation	-0.010 ± 0.061		0.873		-0.130		0.111	Time	0.044 ± 0.034	0.200	-0.023	0.111
*Sleep Disturbance*								Graduation	0.102 ± 0.074	0.172	-0.044	0.248
Time	-0.214 ± 0.047		<0.001^*^		-0.306		-0.122	*Non-Judgement*				
Graduation	0.020 ± 0.083		0.813		-0.144		0.184	Time	0.069 ± 0.043	0.105	-0.014	0.153
**Acceptance**								Graduation	-0.006 ± 0.081	0.936	-0.165	0.152
*Activity Engagement*								*Non-Reactivity*				
Time	0.825 ± 0.051		<0.001^*^		0.725		0.926	Time	0.127 ± 0.038	< 0.001^*^	0.052	0.202
Graduation	-0.037 ± 0.094		0.692		-0.223		0.148	Graduation	0.079 ± 0.062	0.203	-0.043	0.201
Race	0.258 ± 0.107		0.017		0.047		0.469	*Observation*				
*Pain Willingness*								Time	0.137 ± 0.036	<0.001^*^	0.066	0.208
Time	0.304 ± 0.049		< 0.001^*^		0.208		0.400	Graduation	0.038 ± 0.070	0.587	-0.100	0.176
Graduation	-0.044 ± 0.078		0.568		-0.197		0.108					

* Statistically significant (Bonferroni-adjusted *p* < .0025)

*CI* = Confidence Interval; *LB* = Lower Bound; *UB* = Upper Bound; *SE* = Standard Error; HRQoL = Health-Related Quality of Life

Reference Categories: Time (Baseline)^a^, Graduation (Non-Graduate)^b^, Race (Non-White)

##### Main effects of time and graduation

The main effect of time was indicative that PROs significantly changed from baseline to post-EVP. Estimated marginal means and effect sizes for PROs at each time point are reported (see table, Supplementary File 1). Pain (Primary): Participants reported decreases for all three pain-related PROs (*p* < .001) including intensity (*SMD* = − 0.199), interference (*SMD* = − 0.448), and catastrophizing (*SMD* = − 0.392) with effect sizes in the small-to-medium range (see plots, Supplementary Files 2–4). Physical: Participants’ fatigue (*p* < .001, *SMD* = -.300) decreased from baseline to post-EVP, but surprisingly so did their physical functioning scores (*p* < .001, *SMD* = -.175). Both effect sizes were small. Psychological: Participants’ anxiety (*SMD* = − 0.233), depression (*SMD* = − 0.430), and sleep disturbance (*SMD* = − 0.218) scores all decreased from baseline to post-EVP (*p* < .001) indicating small-to-medium effect sizes. Social: Participants social health scores unexpectedly decreased (*p* < .001, *SMD* = − 0.260) from baseline to post-EVP indicating a small effect. HRQoL: Physical (*SMD* = 0.417), psychological (*SMD* = 0.279), social (*SMD* = 0.260), and environmental (*SMD* = 0.188) HRQoL scores all increased from baseline to post-EVP (*p* < .001). Effect sizes in the small-to-medium range. Acceptance: Participants reported increased engagement in life activities despite pain (*p* < .001, *SMD* = 0.779) indicating a large effect. They also experienced a small effect size improvement in willingness to experience pain without attempts to control it (*p* < .001, *SMD* = 0.320). Mindfulness: For FFMQ mindfulness domains participants experienced small effect size improvements in non-reactivity to negative thoughts/emotions (*p* < .001, *SMD* = 0.178) and observing their experiences (*p* < .001, *SMD* = 0.173). This was not the case for describing their own experiences (*p* = .200), non-judgement scores (*p* = .105), and acting with awareness in the moment (*p* = .014), with the latter effect not meeting the Bonferroni-adjusted significance threshold. With the exception of the decrease in physical functioning and social scores, this pattern of significant main effects reflects positive improvements from baseline to post-EVP. Contrary to expectations, there were no significant graduation effects in these in any of the LMMs after the Bonferroni-correction was applied (*p* = .032-0.998).

##### Main effects of race and gender

Main effects of race are indicative of PROs differing between white and non-white participants. Race and gender-based main effects were considered exploratory and dropped from LMMs when non-significant. Psychological: Gender was a non-significant predictor of sleep disturbances (*p* = .085) and dropped from this LMM. Non-white participants (*m* = 2.839) reported lower anxiety scores than white participants (*m* = 3.102), *p* = .011. Social: Non-white participants (*m* = 3.598) PROMIS-29 scale scores indicated lower social health than white participants (*m* = 3.788), *p* = .034. HRQoL: Non-white participants (*m* = 11.983) had higher psychological HRQoL scores than white participants (*m* = 11.186), *p* = .016. Acceptance: On average, non-white participants (*m* = 3.007) had activity engagement scores that were higher than white participants (*m* = 2.749), *p* = .017. Mindfulness: FFMQ acting with awareness scores were higher among non-white participants (*m* = 3.216) than white participants (*m* = 2.982), *p* = .009. Using a more traditional significance threshold (*p* < .05) would have yielded a pattern of race-based main effects that typically indicated more favorable PROs for non-white than white participants. However, none of these race effects met criteria for statistical significance using the more conservative Bonferroni-adjusted threshold (*p* = .0025).

## Discussion

The national response to the opioid epidemic and veteran suicide demands effective non-pharmacological treatments to alleviate chronic pain for veterans. Innovations such as EVP are emerging to support chronic pain management for this priority population. Data is needed to determine the effects of such programs on a spectrum of outcomes. Preliminary data indicate EVP has significant positive outcomes on pain, physical, psychological, HRQoL, acceptance, and mindfulness measures for Veterans with chronic pain. Interestingly, there were no significant graduation improvements which is best explained by the non-granular, binary nature of graduation data and possible variance captured by other model variables (e.g., Acceptance, Mindfulness, HRQoL) that are part in parcel to EVP attendance and ultimately graduation. Our study design and analyses did capture overall health effects, however the weekly attendance of ACT, MM, and WH, each a 1-hour session for 3 hours per week were not recorded. Hence, these data warrant further investigation into the potential dosing effect of the specific EVP components beyond a simple binary measure of program completion. While the effect size was small, it is notable that results indicated that higher age and non-white race were significantly associated with increased likelihood of completing EVP (i.e., graduation). Though satisfaction scores had a wide range (3.2–9.8), the median and IQR indicate that scores were high and did not differ between EVP graduates versus non-graduates. These findings provide some indication that the program is acceptable and useful for diverse audiences.

Results suggest PROs significantly changed from baseline to post-EVP. Primary pain outcomes (pain intensity, interference, catastrophizing) decreased from baseline to post-EVP with medium effect sizes improvement being observed for pain interference and catastrophizing and a small effect for intensity. Using the effect size criteria from the current study, findings from other interdisciplinary chronic pain rehabilitation programs in the VA system found medium (catastrophizing) to large (intensity, interference) within-participant effect sizes. [27] A separate study examining the VA inpatient chronic pain rehabilitation program found a similar pattern of significant effects with these primary outcomes, but effect sizes were not reported for comparison. [31] Of note, the former findings [27] were aggregated across inpatient and outpatient programs that typically operated on cognitive behavioral-based models. Thus, it is a non-equivalent comparison.

Results also indicated positive outcomes on most secondary measures. Physical (fatigue), psychological (anxiety, depression, sleep disturbance), and HRQoL measures (environmental, physical, psychological, social, environmental) improved from baseline to post EVP. Moreover, we observed notable improvements in acceptance (activities engagement, pain willingness) and mindfulness (non-reactivity, observation) measures which provide support for the theoretical foundations of EVP (i.e., ACT, MM). A meta-analysis of group ACT for chronic pain indicated small-to-large between-participant effect size improvements for overall pain acceptance post-treatment versus a heterogenous array of control conditions. [49] The current study examined acceptance as two CPAQ sub-scales rather than as a total score. While, the observed within-participant large effect for activity engagement and small effect size for pain willingness were similar in magnitude to previous findings, the pre-post *SMD* comparison differs from the between-group designs. We were able to make a comparison with EVP and CBT for chronic pain for depression and HRQoL sub-domains. Murphy and colleagues [50] found large within-participant improvements for depression and physical HRQoL as well as medium effects for psychological, social, and environmental HRQoL from pre-post CBT for chronic pain. The current study observed medium within-participant effect size improvements for depression and physical HRQoL as well as small effects for psychological, social, and environmental HRQoL. While the CBT study found larger effect sizes in a VA sample, these interventions vary by their primary theoretical foundation (i.e., CBT v. ACT).

There were unanticipated observed decreases in PROMIS-29 physical functioning and social subscale scores. These findings were seemingly contradictory to observed improvements in physical and social HRQoL. This seems to be an anomaly from this preliminary data sample but warrants further investigation in larger subsequent trials. Collectively findings indicate the holistic benefit of the interdisciplinary approach of EVP.

Though findings are favorable, several limitations should be considered when interpreting results. First, these data represent a limited sample from a single VA facility in an urban setting within the southeast. Additional data should be examined as EVP expands to additional VA sites. Though the sample represents a predominantly urban population, the fairly recent transition to remote delivery of TelePain-EVP will also increase veteran engagement in rural areas. Second, dosing of EVP using attendance data from participation for each of EVP’s subcomponents (ACT, MM, WH) is needed; current data collection efforts are underway. Third, as EVP is a clinical quality management effort, recruitment was based on the pragmatic referral approach rather than randomization, representing a potential self-selection bias, and a referral bias, as mental health providers were responsible for most EVP referrals. Moreover, veterans referred to comprehensive Step 3 pain care programs (i.e., EVP) typically have not responded well to previous steps of pain care (e.g., primary care, specialty pain care) and present with additional complex issues (e.g., depression, opioid use, possible suicidality secondary to pain). [51] Thus, identifying an adequate control group for this unique population is very difficult and under certain circumstances unethical (e.g., suicidality). Fourth, confounding factors were not fully addressed, including evolving guidance for chronic pain management (e.g., decreasing trend in opioid prescriptions), and potentially concurrent services (e.g., CBT, WH/Complementary and Integrative Healthcare services). Fifth, self-report bias which is intrinsic to PRO measures is another limitation of this study. Future studies should integrate objective outcome measures to validate these findings.

Future efforts will benefit from emphasis a larger multi-site sample within the expansion of EVP. Replicability of EVP at additional VA facilities can now be evaluated as the program is being implemented at multiple VA facilities. Subsequent evaluations should include long-term re-assessment of PRO measures, as well as balancing measures (e.g., balanced scorecard or dashboard) to assess other domains to assess unintended consequences. In the response to the COVID-19 pandemic, the VA prioritized expanding veteran access to telehealth services, most recently with Tele-pain services. [52, 53] As such, the potential impact of the recent implementation of TelePain-EVP as a remotely delivered telehealth program is currently being evaluated. To build on current knowledge, future evaluation efforts will also assess dosing effects, the relationship between EVP engagement and medication use, and survival analyses - given the risk of this population for morbidity and mortality, including suicidality. [10, 51]

## Conclusions

As healthcare systems follow the VA’s lead in seeking non-pharmacological treatments to alleviate chronic pain, interdisciplinary programs such as EVP are emerging to support these efforts. Preliminary data suggest Veterans with chronic pain who participate in the EVP program report significant positive outcomes in pain, physical, psychological, HRQoL, acceptance, and mindfulness measures. Though findings are compelling, future evaluations should focus on evaluating larger sample datasets, dosing effects, and effectiveness of the program using a telehealth model to inform ongoing implementation and spread of the EVP program.

## Electronic supplementary material

Below is the link to the electronic supplementary material.

Supplementary Material 1

Supplementary Material 2

Supplementary Material 3

Supplementary Material 4

## Data Availability

The datasets developed and/or analyzed during the current project available from the corresponding author on reasonable request.

## List of Abbreviations

ACT: Acceptance and Commitment Therapy
ANOVA: Analysis of Variance
CARA: Comprehensive Addiction and Recovery ACT
CBT: Cognitive-Behavioral Therapy
CI: Confidence Interval
CPAQ: Chronic Pain Acceptance Questionnaire
EVP: Empower Veterans Program
FFMQ: Five-Facet Mindfulness Questionnaire
HRQoL: Health-Related Quality of Life
IQR: Interquartile Range
LMMs: Linear Mixed Models
MDN: Median
MILES: Motion is Lotion Exercises
MM: Mindful Movement
OR: Odds Ratio
POQ-VA: Pain Outcomes Questionnaire–Veterans Affairs
PCS: Pain Catastrophizing Scale
PEG: Pain, Enjoyment, and General Activity Scale
PHQ-9: Patient Health Questionnaire-9
PMOP: Offices of Rural Health and Pain Management, Opioid Safety, and PDMP
PRO: Patient-Reported Outcomes
PROMIS: Patient-Reported Outcome Measurement Information System
SMD: Standardized Mean Difference
VA: Department of Veterans Affairs
WH: Whole Health
WHOQoL-BREF: World Health Organization Quality of Life Scale
