# Supplementary material for: Empower Veterans Program (EVP): a chronic pain management program demonstrates positive outcomes among veterans
Source: BMC Health Serv Res. 2023 May 3;23:431. doi: 10.1186/s12913-023-09327-5 (PMC10155644; doi:10.1186/s12913-023-09327-5)
Supplement: Supplementary file 1 — Supplementary Material 1 [file 12913_2023_9327_MOESM1_ESM.docx]

Supplemental Digital File 1. Estimated marginal means and 95% confidence intervals for primary and secondary patient-reported outcomes at baseline and post- the Empower Veterans Program (EVP).

| Scale | Time | | | | |  |
| --- | --- | --- | --- | --- | --- | --- |
|  | Baseline | | Post-EVP | | | *SMD* |
|  | Mean | *95% CI*  *LB, UB* | | Mean | *95% CI*  *LB, UB* |  |
| **Pain** |  |  | |  |  |  |
| Intensity^*^ | 7.108 | 6.975, 7.242 | | 6.803 | 6.657, 6.950 | -.199 |
| Interference^*^ | 4.026 | 3.496, 4.106 | | 3.629 | 3.538, 3.721 | -.448 |
| Catastrophizing^*^ | 2.501 | 2.405, 2.597 | | 2.097 | 1.991, 2.202 | -.392 |
| **Physical** |  |  | |  |  |  |
| Fatigue^*^ | 3.665 | 3.576, 3.754 | | 3.369 | 3.268, 3.470 | -.300 |
| Physical Functioning^*^ | 3.489 | 3.412, 3.566 | | 3.340 | 3.254, 3.425 | -.175 |
| **Psychological** |  |  | |  |  |  |
| Anxiety^*^ | 3.102 | 2.989, 3.216 | | 2.853 | 2.729, 2.976 | -.233 |
| Depression^*^ | 1.779 | 1.715, 1.843 | | 1.464 | 1.394, 1.534 | -.430 |
| Sleep^*^ | 3.841 | 3.753, 3.930 | | 3.628 | 3.527, 3.728 | -.218 |
| **Social** |  |  | |  |  |  |
| Social Health^*^ | 3.810 | 3.715, 3.905 | | 3.576 | 3.472, 3.680 | -.260 |
| **HRQoL** |  |  | |  |  |  |
| Physical Health^*^ | 9.040 | 8.803, 9.277 | | 10.172 | 9.908, 10.436 | .417 |
| Psychological Health^*^ | 11.141 | 10.799, 11.483 | | 12.028 | 11.657, 12.398 | .279 |
| Social Health (*t*)^*^ | 37.004 | 34.885, 33.123 | | 43.132 | 40.632, 45.631 | .260 |
| Environmental Health^*^ | 13.071 | 12.798, 13.344 | | 13.640 | 13.333, 13.947 | .188 |
| **Acceptance** |  |  | |  |  |  |
| Activity Engagement^*^ | 2.465 | 2.350, 2.581 | | 3.291 | 3.164, 3.417 | .779 |
| Pain Willingness^*^ | 1.739 | 1.654, 1.823 | | 2.043 | 1.946, 2.140 | .320 |
| **Mindfulness** |  |  | |  |  |  |
| Acting | 3.055 | 2.962, 3.148 | | 3.144 | 3.044,3.244 | .103 |
| Describing | 3.148 | 3.071, 3.225 | | 3.191 | 3.107, 3.276 | -.052 |
| Non-Judgement | 3.012 | 2.927, 3.097 | | 3.082 | 2.987, 3.176 | .076 |
| Non-Reactivity^*^ | 2.848 | 2.781, 2.915 | | 2.975 | 2.898, 3.051 | .178 |
| Observation^*^ | 3.257 | 3.183, 3.330 | | 3.393 | 3.311, 3.475 | .173 |
|  |  |  | |  |  |  |

^*^Significant pre-post EVP change (*p* < .05).

*CI* = Confidence Interval; HRQoL = Health-Related Quality of Life; *IQR* = Interquartile Range; *LB* = Lower Bound; *UB* = Upper Bound; *mdn* = median; SMD = Standardized Mean Difference.
