# Supplementary figures and images for: Empower Veterans Program (EVP): a chronic pain management program demonstrates positive outcomes among veterans
Source: BMC Health Serv Res. 2023 May 3;23:431. doi: 10.1186/s12913-023-09327-5 (PMC10155644; doi:10.1186/s12913-023-09327-5)

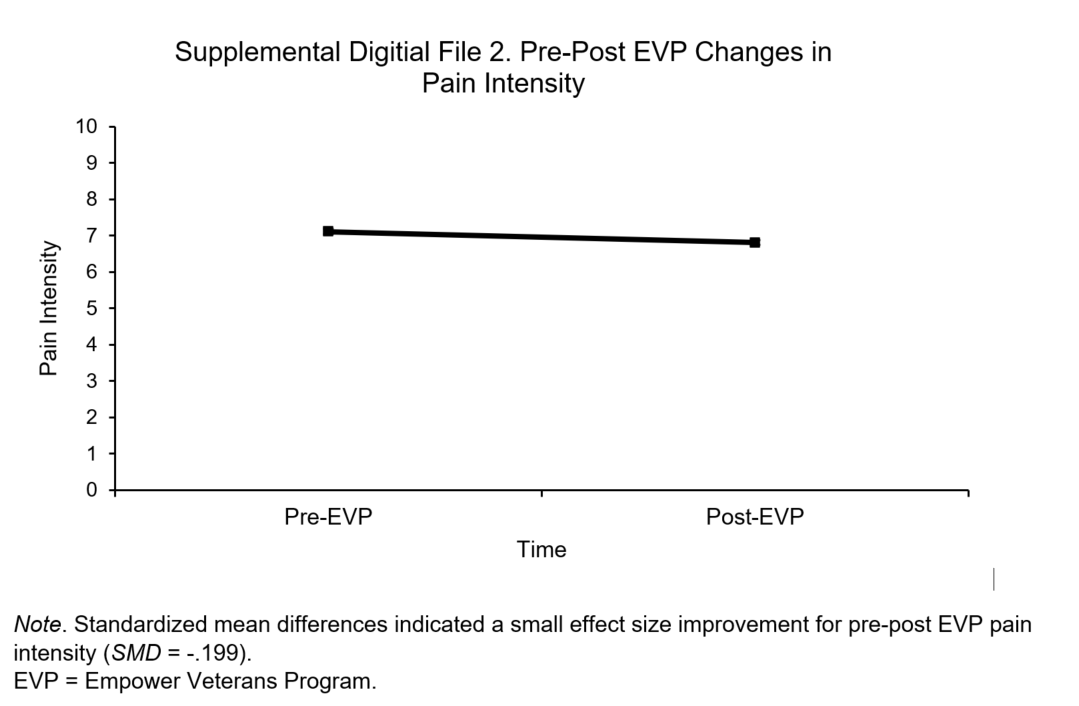

Supplement: Supplementary file 2 — Supplementary Material 2 [file 12913_2023_9327_MOESM2_ESM.png]

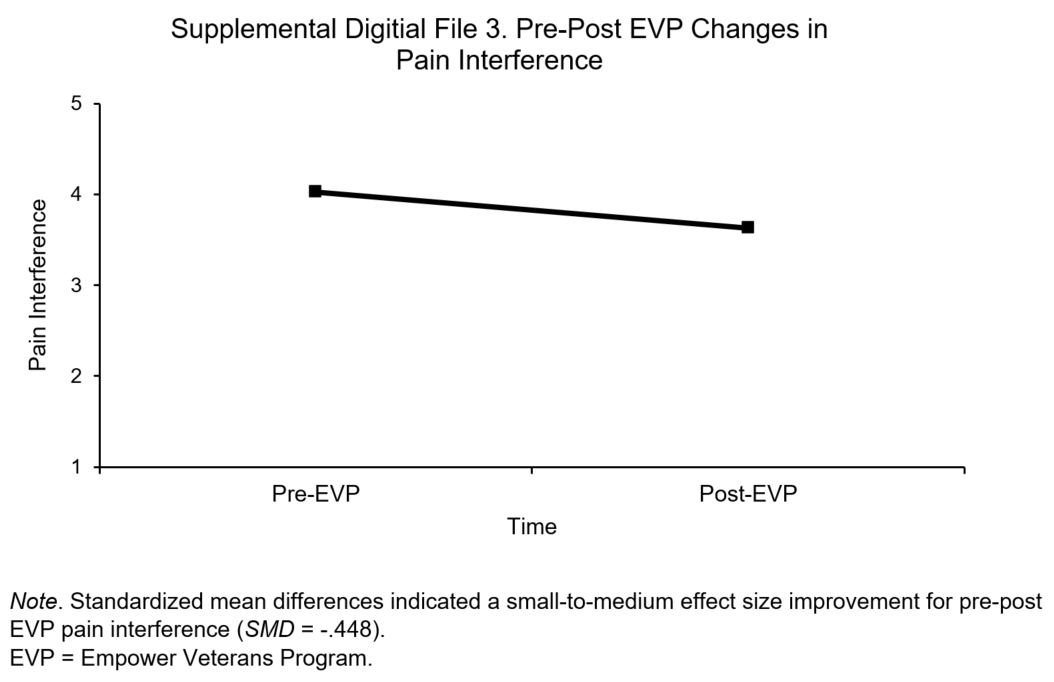

Supplement: Supplementary file 3 — Supplementary Material 3 [file 12913_2023_9327_MOESM3_ESM.png]

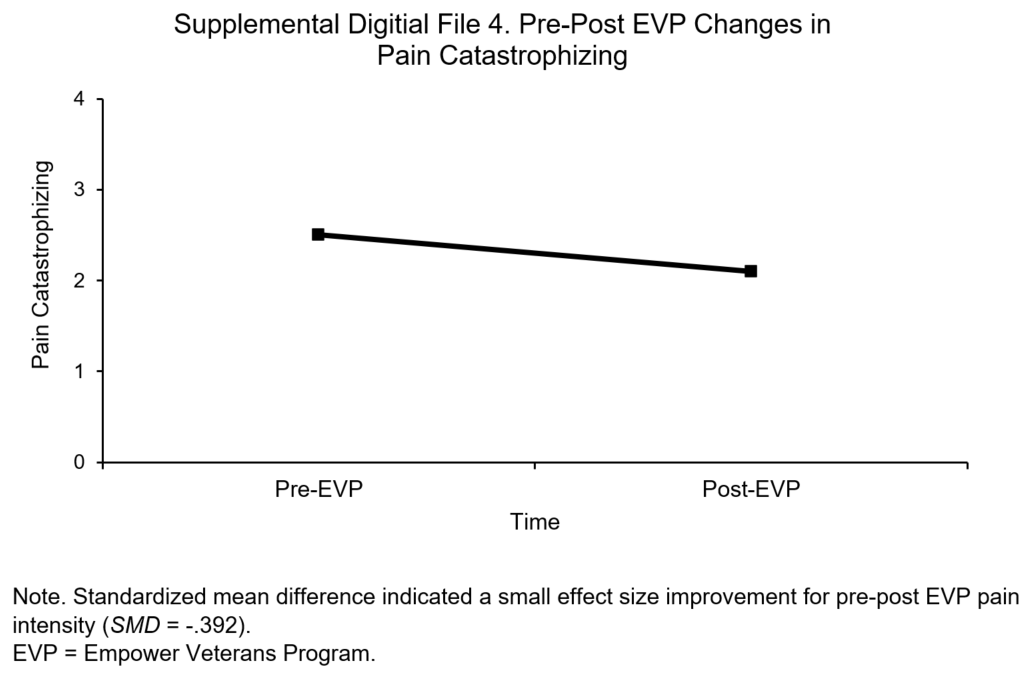

Supplement: Supplementary file 4 — Supplementary Material 4 [file 12913_2023_9327_MOESM4_ESM.png]
